# Measuring educational neglect using the Q method: A model based on the burden of disseminated tungiasis

**DOI:** 10.3389/fepid.2022.1003102

**Published:** 2022-12-09

**Authors:** Ana Carolina Tardin Martins, Luciana Pereira Freire Martins, Renata Velozo Timbó, Natanael Victor Furtunato Bezerra, Ada Amalia Ayala Urdapilleta, Florival Martins Passos Filho, Ciro Martins Gomes

**Affiliations:** ^1^ Programa de Pós-Graduação em Ciências Médicas, Universidade de Brasília, Brasília, Brazil; ^2^ Programa de Pós-Graduação em Patologia Molecular, Universidade de Brasília, Brasília, Brazil; ^3^ Secretaria de Saúde do Distrito Federal, Brasília, Brazil; ^4^ Instituto de Gestão Estratégica de Saúde do Distrito Federal, Brasília, Brazil

**Keywords:** clinical education, community based education, instructional materials/methods, primary care education, qualities/Skills/Values/Attitudes. 2

## Abstract

**Background:**

A paramount factor in the control of neglected tropical diseases from both medical and social aspects is education. New strategies must be constantly pursued to test and provide educational information related to diseases affecting vulnerable populations. We applied the Q method as a model to measure educational neglect based on the burden of disseminated tungiasis.

**Methods:**

Using a saturation method for sample size calculation, we recruited students and healthcare professionals to evaluate and classify 27 statements related to the prevention, control and treatment of tungiasis. After quantitative analysis, the Q method was applied based on the paired use of the centroid method and Varimax rotation, and 4 factors were extracted representing the main sets of viewpoints among the participants.

**Results:**

We included 119 healthcare professionals with different academic degrees. Statements classified by specialists with a + agreement were also classified as a + agreement by most of the participants. However, we detected 5 important disagreements related to the topical treatment of tungiasis and control of the disease in the environment and animals. The Q method showed that almost no consensus was detected for four statements. The classification of each statement was not related to the participants' academic degree.

**Conclusions:**

There is significant educational neglect related to tungiasis prevention and treatment in healthcare sciences in Brazil. We conclude that the Q method may be an interesting strategy alone or associated with quantitative strategies for detecting educational limitations related to neglected diseases. In countries where neglected diseases are endemic, a detailed study evaluating the quality of education related to these diseases must be prioritized.

## Introduction

Neglected diseases are relevant public health problems, and the control of these diseases is hampered by a lack of investment by and attention from public and private sectors (1, 2). Although scientific advancements have been achieved with technology investment and globalization, some emerging and re-emerging neglected tropical diseases are growing problems that mainly affect individuals in developing countries but pose serious problems to those in developed areas as well (3). In some communities, located in Central and South America and in Africa, tungiasis has a high prevalence of more than 50%, but the disease is easy to diagnose and treat. Therefore, control campaigns would be worthwhile. Some recent phenomena such as global warming and intense migration flows demonstrate that controlling these diseases is important for all countries (2).

Tungiasis is not only a problem of the individual, but of the entire community (4). Although neglected tropical diseases have a direct impact on public health in the form of high levels of morbidity and mortality, the elevated and frequently unmeasured indirect costs of these diseases are also substantial (5). This indirect and unpredictable effect is related to many areas and generates social, educational, psychological, nutrition, work and pension system impacts. These impacts are difficult to measure. However, education is a paramount factor in controlling neglected tropical diseases from both medical and social aspects; specifically, populational and professional education are the key to effective prevention and therapeutic interventions for mitigating these diseases. New strategies must be constantly pursued to test and provide educational information related to vulnerable populations.

Tungiasis is a neglected disease caused by the female sandflea *Tunga penetrans*, which is present in sandy soils and infects individuals by penetrating unprotected human skin (6, 7). It is a common disease in developing countries and can usually be mitigated by basic environmental actions including education, physical protection and the use of repellents such as zanzarin (8, 9). However, disseminated cases can disproportionately affect those in poor communities, leading to patients with more than 100 lesions and resulting in secondary infections and deformities (8, 10). Most people are unaware that tungiasis can progress to such severe outcomes, but these cases have been found in remote communities in South America and Africa (10). Thus, tungiasis is an interesting model for testing the relationship between the various academic degrees of participants and the educational neglect in countries where the disease is endemic.

The aim of the present study was to apply the Q method as a model for measuring educational neglect related to tungiasis based on previous information collected from systematic reviews of the literature. We also aimed to create recommendations for educational policies targeting healthcare providers involved in the fight against disseminated tungiasis.

## Methods

First, 2 medical doctors (AM and CG) involved in treating tungiasis in daily clinical practice and disseminated tungiasis in vulnerable communities created 27 statements about the relevance of the disease (Table 1). The statements covered important aspects of the disease, including the relevance of the disease for public health, diagnosis, prevention, treatment and associated morbidities. Each statement was initially classified by the two specialists as true or false for future comparison. Responses to the statements were measured on a scale from −5 to +5, meaning that the greater the score, the more the specialists agreed that the statements were true. The classification of each statement followed the findings reported in 2 comprehensive reviews of the literature evaluating the treatment of tungiasis (8, 11). Localized tungiasis is very common in Brazil and although proper treatment and prevention are neglected, the studied population have basic knowledge related to the main symptoms of the disease.

**Table 1:** The 27 statements assessed along with the specialists’ classification and participants’ pooled quantitative and qualitative analyses (Q method).

Statement number	# Study statement	Specialists’ classification	Mode	Median (range)	**Factor 1** QSort Value ZScore Value	**Factor 2** QSort Value ZScore Value	**Factor 3** QSort Value ZScore Value	**Factor 4** QSort Value ZScore Value
1	Tungiasis affects communities with low social and economic development.	True (+5)	+3	+2 (−4; +5)	+2 0.9040	0 0.2208	+5 1.5583	+3 0.9176
2	Tungiasis is a relevant problem for Brazilian public health.	True (+3)	+3	+2 (−4; +5)	+4 1.2177	+4 1.2616	+2 0.8472	+1 0.3944
3	Tungiasis causes secondary bacterial infections.	True (+3)	+4	+2 (−5; +5)	+3 1.1144	+3 0.9591	+3 1.0656	+3 1.0470
4	Tungiasis predisposes individuals to tetanus.	True (0)	0	+1 (−5; +5)	+3 1.2145	0 0.2168	−2 −0.5737	−2 −0.7494
5	Wearing shoes solves the problem related to tungiasis.	False (−1)	−1	0 (−5; +5)	0 0.1331	−3 −1.2951	−1 −0.0215	+3 1.0735
6	High frequency of tungiasis infestation reflects a community sanitation problem.	True (+3)	+1	+2 (−5; +5)	+1 0.7218	+3 1.1768	+3 1.0223	+5 1.6018
7	Tungiasis is a traveller's disease.	False (−4)	−3	−2 (−5; +5)	−2 −0.8006	−1 −0.6886	−4 −1.5368	−2 −0.7138
8	The spread of tungiasis is favoured by raising domestic animals.	True (+3)	+2	+1 (−5; +5)	0 −0.3545	+5 1.6284	+2 0.6045	0 0.2870
9	The spread of tungiasis is favoured by interaction with wild animals.	False (−5)	−1	−1 (−5; +5)	0 −0.3573	−3 −1.1589	+2 0.7443	−2 −0.9469
10	Manual/surgical extraction of tungiasis is a suitable treatment for tungiasis.	True (+4)	+3	+2 (−4; +5)	+5 1.6503	+4 1.2652	0 0.0537	+2 0.6542
11	Manual/surgical extraction of tungiasis can be done in a residential setting.	False (−5)	−1	−1 (−5; +5)	−2 −0.9272	+1 0.4682	−1 −0.4943	−1 −0.0202
12	Topical medications are suitable treatments for tungiasis.	True (+5)	−3	−1 (−5; +5)	−1 −0.6614	−3 −1.0467	+1 0.4699	+1 0.5810
13	Systemic medications are suitable treatments for tungiasis.	False (−5)	−3	−1 (−5; +4)	−3 −1.0112	−1 −0.0728	−4 −1.3145	0 0.1519
14	Tetanus prophylaxis is a crucial measure for communities with high infestation by *Tunga penetrans*.	True (+5)	+2	+1 (−5; +5)	+3 1.0823	+1 0.5373	−3 −1.2407	−1 −0.2814
15	The treatment of tungiasis should be done in the Basic Health Unit.	True (+3)	+3	+2 (−5; +5)	+4 1.5532	−1 −0.1835	+3 0.9632	+1 0.5744
16	The treatment of tungiasis should be done in a specialized dermatological office.	False (−5)	−1	−2 (−5; +5)	−5 −1.6586	+1 0.3983	−2 −1.0853	−3 −1.1218
17	Traditional systemic and topical options should be researched for the treatment of tungiasis.	True (0)	+1	+1 (−5; +5)	+1 0.2404	0 −0.0544	0 0.3881	+2 0.7403
18	The treatment given to indigenous populations can be the same as that given to other populations affected by tungiasis.	False (−3)	0	−1 (−5; +4)	−1 −0.5841	−2 −1.0132	−2 −0.8721	+2 0.7154
19	The environmental control of tungiasis needs improvements related to basic sanitation.	True (+3)	+3	+3 (−4; +5)	+2 0.9560	+2 0.9365	+4 1.5225	+4 1.2151
20	Systemic treatment of domestic animals is essential for the environmental control of tungiasis.	True (+2)	−2	−1 (−5; +5)	−1 −0.6871	+3 1.0785	+1 0.5539	−3 −1.0492
21	Control of wild animals is essential for the environmental control of tungiasis.	False (−4)	−4	−2 (−5; +5)	−3 −1.0347	−4 −1.3189	+4 1.0748	−4 −1.5400
22	The use of pesticides is viable for the environmental treatment of tungiasis.	True (+1)	−5	−3 (−5; +2)	−4 −1.1972	−5 −1.6772	−5 −1.8713	−5 −1.7008
23	Soil humidification is effective for the environmental control of tungiasis.	True (+2)	−3	−2 (−5; +4)	−4 −1.1101	−4 −1.5916	−3 −1.0962	−1 −0.6696
24	Infrastructure works are decisive for the control of tungiasis.	True (+4)	+4	0 (−5; +4)	+1 0.2868	−2 −0.7232	0 0.2440	0 0.1725
25	Health education is a viable strategy for tungiasis control in housing and indigenous communities.	True (+6)	+2	+2 (−5; +5)	+2 1.0348	+2 0.8645	+1 0.5780	+4 1.5054
26	Prophylactic oral treatment of domestic animals is effective in the environmental control of tungiasis.	True (+1)	−2	−2 (−5; +5)	−2 −0.7927	2 0.6191	−1 −0.4935	−3 −1.3589
27	Prophylactic oral treatment of affected people is effective in the environmental control of tungiasis.	False (−5)	−3	−2 (−5; +5)	−3 −0.9326	−2 −0.8067	−3 −1.0904	−4 −1.4796

### Study population

We recruited anonymous undergraduate and postgraduate students and healthcare professionals from the University of Brasília (UnB), Brazil. The university is responsible for a variety of graduation courses, specializations, and master's degree and PhD courses. The university is also responsible for the University Hospital of Brasília (HUB) and employs a variety of healthcare professionals. Members of the target population were invited to participate using the institutional email system. The purpose of the study, instructions about the survey, requests for information and consent forms were presented on a web-based form appropriate for the present methodology (12).

### Q method

We used the web-based Q-method software (12) for the analysis of the viewpoints of healthcare providers. The Q method is the systematic study of participant viewpoints. It is a form of gathering semiquantitative overviews of qualitative data (13, 14). It helps researchers and policymakers in several areas, including psychology, environmental areas and health science, to generate conclusions related to subjective data (15). The first step in applying this method was the creation of the target statements as previously described.

**Figure 1:**
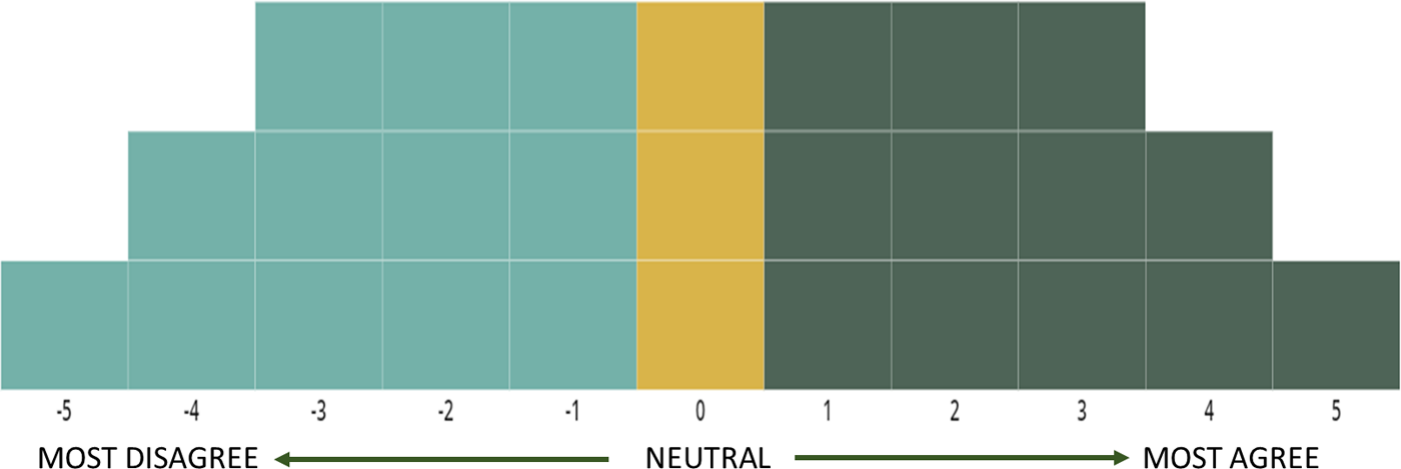
Q-sorting grid used in the present research. The wide pyramid base was constructed to reduce the number of neutral classifications.

Each participant was instructed to read and classify each statement according to the following opinions: agree, neutral, disagree. The responses were then assessed by the Q-sort process. Each statement was placed inside a box according to a previously defined pyramid structure (Figure 1). Each column represented an opinion: 0 (yellow boxes) = neutral; −1 to −5 (light green boxes) = disagree; +1 to +5 (dark green boxes) agree. In this step, agreements and disagreements were classified in a quantitative way according to the participants' opinions. The statements with the most agreement were placed, as much as possible, in the right part of the Q-sort pyramid (Figure 1). It is important to note that the statement sorting process is influenced by the pool of statements representing the importance of each phrase in the studied field and that this process is designed to provide important information about what should be prioritized in the future.

### Data analysis

The demographic data of each participant and the individual answers to each statement (from −5 to +5) were analysed by classic quantitative methods. Comparisons of categorical variables were made using the chi-squared test or its exact version depending on the frequency of each occurrence. Descriptive analysis of the classifications attributed by each participant to each statement involved calculating the mode, median and interquartile range values. As one of the main objectives of the study was to test aspects related to the educational neglect regarding tungiasis, we included an analytical evaluation of participants' statement classifications as they relate to their academic degree. We used Wilcoxon tests for numerical values depending on the nature and distribution of data. Quantitative statistical analysis was performed using program R version 4.1.2 [R Core Team (2021). R: A language and environment for statistical computing. R Foundation for Statistical Computing, Vienna, Austria. URL https://www.R-project.org/]. Statistical significance was defined as a *p* value <0.05 and a 95% confidence interval (CI).

The analysis using the Q-method software (12) relied on the creation of a correlation matrix using the Pearson method. The matrix compares the individual responses of all participants to test if and how they correlate. Subsequently, we used strategies to identify patterns in participants' answers. Those patterns were denominated factors. Factors can also be classified according to the capacity of separating different opinions and are considered tendencies of those opinions found in different sorts of that same population. For this purpose, we used the centroid extraction method wherein the research was based on the *a priori* hypotheses that tungiasis is a neglected disease and that participants would probably not agree with assumptions stating that tungiasis is a relevant public health problem and that the disease is associated with significant morbidity. We opted to extract 4 factors, representing the 4 most important sets of different viewpoints from the participants. Subsequently, we used the Varimax method to perform factor rotation (16). The paired use of the centroid extraction method and Varimax rotation is the most accepted methodology in publications for exploratory analysis (12, 17).

In the final step of the analysis, based on the statistical background described, the distinguishing statements for each of the 4 factors were accessed (12). A distinguishing statement occurs when participants classify that statement in a significantly different manner than all the other viewpoints. When there are no differences between any pair of factors, the participants are considered to be in consensus regarding that statement. Additionally, we labelled each statement as most characteristic, most uncharacteristic, quite characteristic and quite uncharacteristic depending on the pooled classification of each statement in each factor.

### Sample size

**Figure 2:**
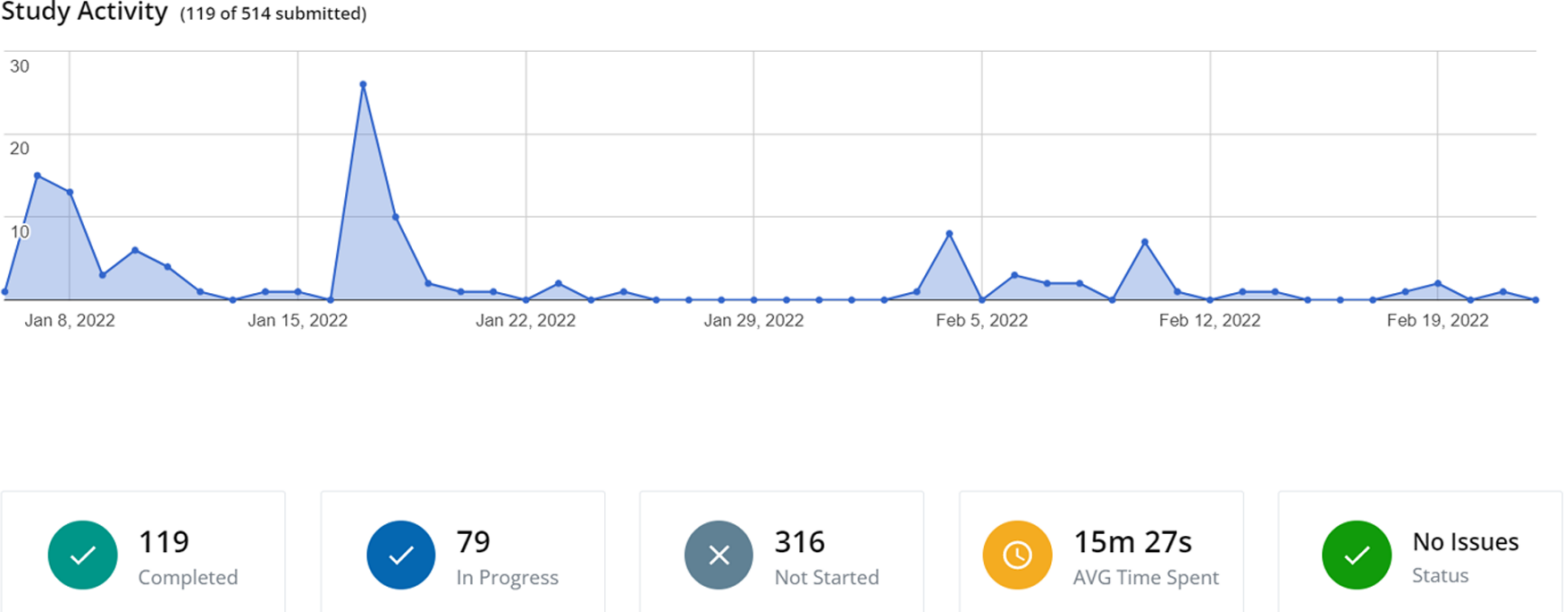
Study recruitment activity and information related to the participants.

The sample size of our study was based on the principle of data saturation, the most commonly used method for qualitative research (18). This method relies on the assumption that when no new data and no new information are added to the study, there is a great probability that the target population was achieved and that any new efforts will probably be unfeasible (18). Recruitment began on January 6th, 2022, using the institutional email system. On January 15th, a reminder was sent to the target population, and this action was shown to be effective for recruitment. Two additional reminders were sent on February 5th and February 12th. Ten days later, with evidence of no new data in the pool and considering that recruitment achieved a population number sufficient for the assumption of normality, we interrupted the research inclusion process. Study recruitment activity is shown in Figure 2.

### Ethics

The participants were included after signing an informed consent form. The present research was approved by the Ethics Committee of the Faculty of Medicine, Universidade de Brasília (UnB) (CAAE: 53039921.3.0000.5558). All methods were carried out in accordance with relevant guidelines and regulations.

## Results

One hundred nineteen participants successfully completed the survey. Most of the participants (74) were females, and the mean age of the participants was 33.82 years. Thirty-two participants were undergraduate students of medicine, 16 had graduated in health sciences (graduation course: 9 medicine, 2 dentistry, 1 biomedicine, 1 nursing, 1 physical therapy, 1 audiology and 1 biomedical engineering), 24 had specialized (specialization-related areas: 13 medicine, 6 nursing, 3 pharmacy, 1 nutrition, 1 psychology), 27 had a master's degree, and 20 had a PhD. Due to the great variability in academic degrees, especially regarding non-academic specialization, we did not perform a quantitative statistical analysis comparing those groups.

In our quantitative analysis, we observed that most statements classified by specialists with a + agreement were also classified as a + agreement by most of the participants, considering the mode and median values (Table 1). However, there was an evident disagreement between the specialists in tungiasis and the other participants regarding the following 5 assumptions: 12: topical medications are suitable treatments for tungiasis, 20: systemic treatment of domestic animals is essential for the environmental control of tungiasis, 22: the use of pesticides is viable for the environmental treatment of tungiasis, 23: soil humidification is effective for the environmental control of tungiasis, and 26: prophylactic oral treatment of domestic animals is effective in the environmental control of tungiasis. The analysis showed that most of the participants disagreed (attributed a negative value) with those assumptions, while the specialists agreed with them (attributed positive values).

The representativeness of each statement for the 4 factors extracted by the selected Q-method software is presented in Table 2. The only statement that resulted in a consensus for all 4 extracted factors with a classification of +3 was statement number 3: tungiasis causes secondary bacterial infections (Table 2). Additionally, statement number 19, namely, the environmental control of tungiasis needs improvements related to basic sanitation, resulted in a consensus for factor 4 with a classification of +4 (Table 2).

**Table 2:** Classification of the statements according to the Q method. The 4 factors extracted represent the 4 main pools of opinions in the total group of participants included in the study.

	Factor 1	Factor 2	Factor 3	Factor 4
Distinguishing statements	10, 15, 4, 14, 8, 9, 12, 20, 11, 22, 126	8, 10, 20, 26, 14, 11, 16, 1, 4, 15, 24, 12, 5, 23	1, 21, 15, 2, 9, 20, 10,11, 14, 7	6, 25, 5, 18, 10, 15, 2, 11, 14, 23, 20, 26, 27
Consensus statements	3	3	3	19, 3
Most characteristic	10, 15, 2, 4, 3, 14	8, 10, 2,6, 20, 3	1, 10, 21, 3, 6, 15	6, 25, 19, 5, 3, 1
Most uncharacteristic	27, 13, 21, 23, 22, 16	12, 9, 5, 21, 23, 22	26, 23,14,13,7, 22	20, 16, 26, 27, 21, 22
Quite characteristic	25, 19, 1, 6, 24, 17	19, 25, 26, 14, 11, 16	2, 9, 8, 25, 20,12	17, 18, 10, 12, 15, 2
Quite uncharacteristic	18, 12, 20, 26, 7, 11	13, 15, 7, 24, 27, 18	5, 26., 11, 4,18, 16	11, 14, 23, 27, 4, 9

The median classification of each statement was not related to the participants' academic degree in most cases (Table 3). Although the academic degree apparently influenced statements 1 (Tungiasis affects communities with low social and economic development.), 8 (The spread of tungiasis is favoured by raising domestic animals.) and 9 (The spread of tungiasis is favoured by interaction with wild animals.), no pattern that could justify the influence of the evaluated academic degree on the knowledge of tungiasis was found (Table 3).

**Table 3:** Analytical assessment of the participants’ responses according to their academic degree.

Statement Median (IQR)	Undergraduate^a^	Graduate^b^	Specialized^c^	Master's Degree^d^	Doctoral Degree^e^	*P* value (Significant Differences)
Statement 1	+3.00 (2.00)	+2.00 (1.25)	+1.50 (2.25)	+1.00 (4.00)	+2.50 (1.25)	0.046 (d × e)
Statement 2	+3.00 (3.00)	+2.00 (5.25)	+1.00 (4.00)	+2.00 (3.00)	+2.00 (5.00)	0.457
Statement 3	+2.50 (3.00)	+1.50 (2.50)	+2.00 (3.00)	+2.00 (2.50)	+2.50 (2.25)	0.371
Statement 4	+0.50 (3.00)	+1.50 (4.00)	+1.00 (3.00)	0.00 (3.50)	+1.00 (4.00)	0,696
Statement 5	−1.00 (4.00)	−0.50 (3.25)	0.00 (4.25)	+1.00 (3.50)	0.00 (4.00)	0.763
Statement 6	+2.50 (3.25)	+1.50 (3.25)	+2.00 (2.00)	+2.00 (3.00)	+2.00 (2.50)	0.985
Statement 7	−2.00 (3.00)	−1.00 (3.00)	−2.50 (2.25)	−3.00 (4.00)	−2.00 (4.25)	0.366
Statement 8	−1.00 (3.00)	0.00 (3.25)	+1.00 (3.00)	+2.00 (2.50)	0.00 (2.25)	<0.001 (a × c; a × d)
Statement 9	0.00 (3.00)	0.00 (3.50)	−1.00 (4.25)	−1.00 (2.50)	−2.00 (2.25)	0.002 (a × d; a × e)
Statement 10	+2.00 (4.00)	+1.50 (2.25)	+2.50 (4.25)	+3.00 (4.50)	+1.00 (4.25)	0.849
Statement 11	−1.00 (4.25)	−1.00 (2.25)	−0.50 (4.00)	−1.00 (3.00)	−1.00 (3.25)	0.555
Statement 12	−0.50 (3.25)	0.00 (4.50)	−2.00 (3.25)	−1.00 (3.50)	−1.00 (4.25)	0.461
Statement 13	−2.00 (3.25)	−2.00 (2.25)	0.00 (2.50)	−1.00 (3.00)	−0.50 (4.00)	0.145
Statement 14	+2.00 (2.00)	+0.50 (5.00)	+1.00 (3.50)	+1.00 (3.00)	+0.50 (2.50)	0.669
Statement 15	+2.00 (3.00)	+2.00 (2.00)	+2.50 (1.25)	+1.00 (4.00)	+3.00 (3.25)	0.688
Statement 16	−3.00 (2.25)	−1.50 (3.25)	−2.00 (2.00)	−2.00 (4.00)	−2.00 (1.00)	0.094
Statement 17	+1.00 (2.00)	+1.00 (3.00)	+0.50 (2.25)	+1.00 (2.00)	+0.50 (3.25)	0.678
Statement 18	−1.00 (4.00)	−1.00 (3.25)	−1.50 (2.25)	0.00 (3.00)	−0.50 (3.25)	0.638
Statement 19	+2.00 (2.00)	+3.00 (2.00)	+3.00 (2.00)	+2.00 (3.50)	+2.50 (2.000)	0.273
Statement 20	−1.50 (3.00)	0.00 (4.00)	0.00 (5.25)	0.00 (4.00)	−1.00 (4.00)	0.0935
Statement 21	−1.50 (2.25)	−2.00 (3.25)	−1.00 (3.25)	−2.00 (3.00)	−3.00 (2.00)	0.120
Statement 22	−3.00 (2.00)	−3.00 (2.00)	−3.00 (3.25)	−4.00 (2.00)	−3.00 (5.00)	0.707
Statement 23	−3.00 (3.00)	−2.00 (1.25)	−3.00 (3.25)	−2.00 (2.00)	−1.00 (2.25)	0.174
Statement 24	+1.00 (3.25)	+0.50 (3.00)	0.00 (4.25)	0.00 (4.00)	−1.00 (3.00)	0.874
Statement 25	+3.00 (2.00)	+1.00 (5.25)	+2.50 (2.00)	+2.00 (3.00)	+2.00 (2.00)	0.080
Statement 26	−2.00 (2.25)	−2.00 (3.25)	−2.00 (2.00)	−1.00 (5.00)	−1.50 (3.00)	0.444
Statement 27	−2.00 (2.00)	−2.00 (3.25)	−3.00 (3.00)	−3.00 (3.50)	−2.50 (2.25)	0.951

## Discussion

Although qualitative methods are constantly evolving, the analysis of data that are not purely quantitative and the transformation of this analysis into recommendations is still difficult (19). The present study relied on an initial quantitative step to analyse the knowledge of healthcare professionals in relation to a relevant public health problem. We used the Q method to gather more direct conclusions from qualitative data (i.e., opinions) collected from those professionals. The Q method is one of the most popular strategies for analysing qualitative data and is considered one of the most interesting forms of gathering information from sources and transforming it into direct conclusions and recommendations (16, 17, 20, 21).

An important step in the successful application of this approach is the selection of an adequate population. As our aim was to test educational properties associated with the successful control of tungiasis, we selected a population comprising students of graduation courses in health sciences and graduated professionals in health sciences. We also recruited participants with different academic degrees in health sciences to allow comparisons. Demographic evaluation of the participants shows that we recruited a sample of healthcare providers in the early stages of their professional career according to the age profile (mean age = 33.82 years), allowing a better evaluation of the formal educational background related to the control of tungiasis. The variety of educational degrees and graduation areas was also ideal for the effect of time on acquisition of current knowledge of tungiasis. Additionally, we considered this an ideal framework for evaluating a disease that is usually treated at the primary care level or at home (22).

Comparing the opinion of participants to the classification from specialists, we detected some important disagreements when comparing positive (+) to negative (−) classifications. The participants disagreed with questions 12 (Q sort = −3), 20 (Q sort = −2), 22 (Q sort = −5), 23 (Q sort = −3) and 26 (Q sort = −2) (Table 1). These results represent an important lack of knowledge about tungiasis control, especially the control of large-scale infestations. Recent systematic reviews and clinical trials have shown that topical treatment with dimethicone compounds was highly effective for the control of tungiasis (8, 11, 23–25). In addition, observational studies have demonstrated that environmental and animal control are essential for the control of tungiasis (8, 26). However, the participants disagreed with these statements, showing a common misconception that tungiasis should be treated only with mechanical extraction. Mechanical extraction in underdeveloped areas can be a source of complications such as secondary bacterial infection, viral hepatitis, human immunodeficiency virus infection and tetanus (8). Since tungiasis is a zoonosis and the maintenance of infection sources depend on the infestation of soil, maintaining control of human infestation in communities and villages depends on a One Health approach (27).

The evaluation of the results using the Q method also points to a relevant gap in the knowledge related to tungiasis in the interviewed healthcare professionals. In the extracted factors, representing the 4 main types of opinions that could be grouped by the methodology used, disagreement was very high. The only consensus associated with 3 of the 4 extracted factors was related to assumption 3, in which healthcare professionals recognize that tungiasis can be complicated by secondary infections. This result points to a lack of formal education in the field of tungiasis in those with different academic degrees and in different areas of health sciences in the educational curriculum in Brazil. Many factors can explain this impressive educational neglect related to tungiasis. The link between tungiasis and poverty is a major obstacle for the knowledge related to tungiasis since the affected population is also neglected. Low public and private investments can also explain the low number of professionals dealing with disseminated tungiasis.

The main limitation of the present study is the small number of professions in multidisciplinary areas of healthcare. Most included participants were from the medical area. Although medical doctors represent an important overview of the Brazilian educational system, tungiasis is a disease related to social issues; thus, a wider spectrum of action for control is necessary. Additionally, this relatively small variety of professions precludes any deep analytical analysis on this aspect. Also, the present sampling method can't avoid the existence of selection bias. Participants that agreed to participate may be interested in the theme discussed in the survey form. It is not possible to generalize the present result to national or international populations.

## Conclusions

We can conclude that there is significant educational neglect related to tungiasis in Brazil, a country in which this disease is endemic. We can also conclude that the Q method may be an interesting strategy alone or associated with quantitative strategies for detecting educational limitations related to neglected diseases. In countries where neglected diseases are endemic, a detailed study to evaluate the quality of education related to those diseases must be prioritized.

## Data Availability

The raw data supporting the conclusions of this article will be made available by the authors, without undue reservation.
